# Cellular and Extracellular Components in Tumor Microenvironment and Their Application in Early Diagnosis of Cancers

**DOI:** 10.1155/2020/6283796

**Published:** 2020-01-08

**Authors:** Rui Wei, Si Liu, Shutian Zhang, Li Min, Shengtao Zhu

**Affiliations:** Department of Gastroenterology, Beijing Friendship Hospital, Capital Medical University, National Clinical Research Center for Digestive Disease, Beijing Digestive Disease Center, Beijing Key Laboratory for Precancerous Lesion of Digestive Disease, Beijing 100050, China

## Abstract

Tumors are surrounded by complex environmental components, including blood and lymph vessels, fibroblasts, endothelial cells, immune cells, cytokines, extracellular vesicles, and extracellular matrix. All the stromal components together with the tumor cells form the tumor microenvironment (TME). In addition, extracellular physical and chemical factors, including extracellular pH, hypoxia, elevated interstitial fluid pressure, and fibrosis, are closely associated with tumor progression, metastasis, immunosuppression, and drug resistance. Cellular and extracellular components in TME contribute to nearly all procedures of carcinogenesis. By summarizing the recent work in this field, we make a comprehensive review on the role of cellular and extracellular components in the process of carcinogenesis and their potential application in early diagnosis of cancer. We hope that a systematic review of the diverse aspects of TME will help both research scientists and clinicians in this field.

## 1. Introduction

The concept of tumor microenvironment (TME) has been proposed for more than one hundred years. In 1889, Stephen Paget proposed the “seed and soil” theory, pointing out that cancer metastases require both the dissemination of cancer cells (the “seed”) and a special affinity for the growth-enhancing milieu of specific organs (the “soil”) [1]. Since then, oncologists have revealed many multiple functions of TME components not only in cancer metastasis and growth but also in cancer metabolism and progression [2].

**Figure 1:**
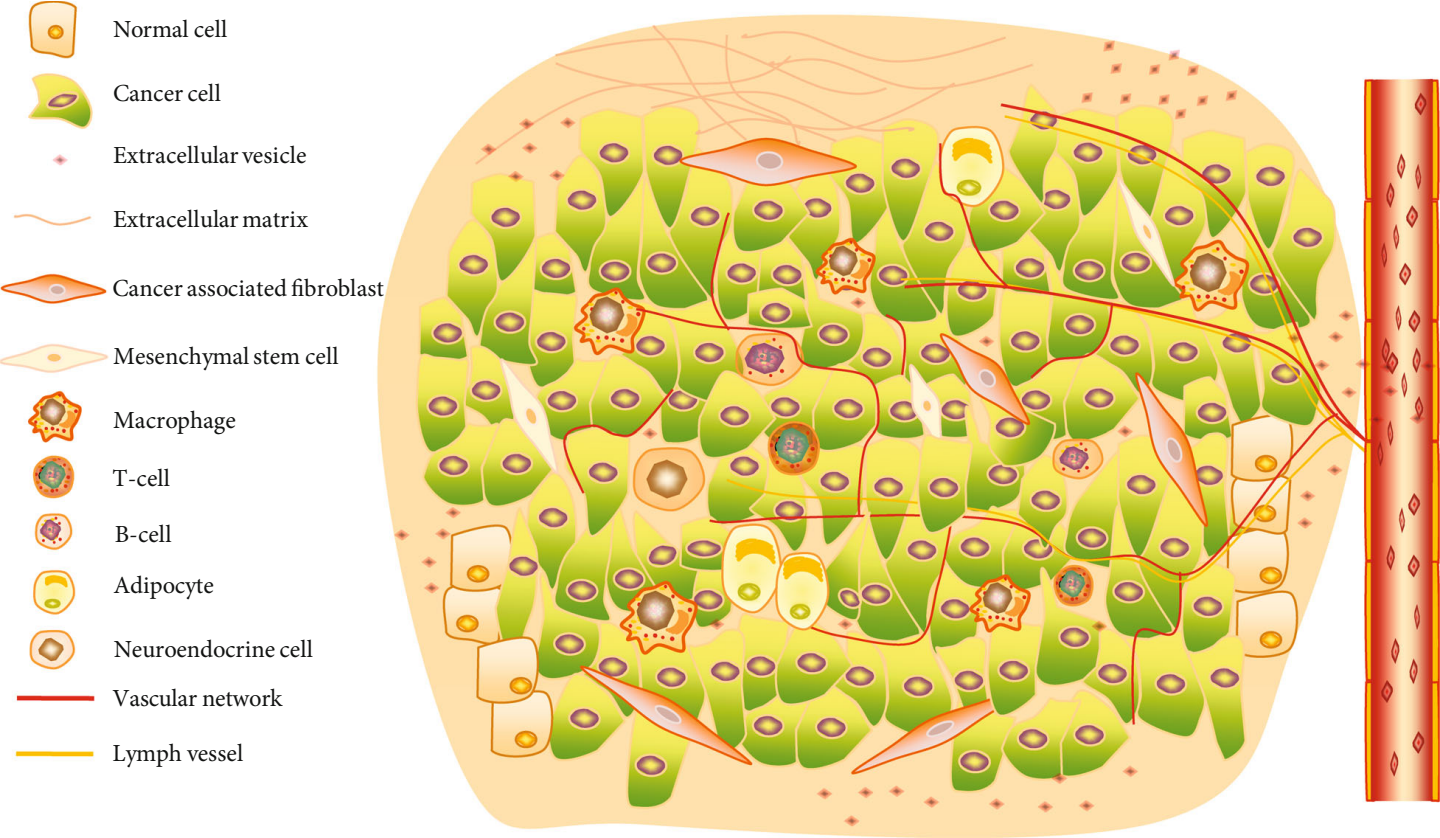
Complex components of the TME. The scheme indicates multiple cellular and other noncellular parts form the web of the TME together.

Tumors are generally highly heterogeneous and complex in genetics. Diverse types of cells, including fibroblasts, endothelial cells, adipocytes, immune cells, and neuroendocrine (NE) cells, have special functions in TME [2, 3] (Figure 1). Acellular components such as the extracellular matrix (ECM), extracellular vesicles (EVs), and cytokines surrounding these cells were also identified [3, 4] (Figure 1). Physical and chemical characteristics of the microenvironment (low pH, hypoxia, high interstitial pressure, and fibrosis) were also included as critical microenvironmental players [5–7]. Besides, interactions between cells and stromal components also play an ever-increasing role in cancer development and progression [4, 8].

In the last decade, new approaches, technologies, and remarkable insights emerged in the fields of cancer biology [9, 10]. More participants and their complex interconnections in TME have been revealed. This review intends to supply some information and recent researches of the components in TME, with a particular focus on their potential application in early diagnosis.

## 2. Role of TME in Cancer Progression: Structure, Cells, and Signaling

TME is a web of cancer-associated fibroblasts, immune cells, extracellular matrix, and vasculature (Figure 1). It is hypothesized that the crosstalk between cancer cells and their surrounding environmental factors plays a pivotal role in tumor development [11]. Intriguingly, each component in TME may play invert roles in early or advanced tumors, which may bring more complicated challenges for cancer therapy. It is hard to assert the helpful or harmful function of TME depending on the disease context. In this part, we will summarize our current understanding of the composition of TME and how they impact cancer biology.

### 2.1. Cancer-Associated Fibroblasts (CAFs)

Among all components in the TME, cancer-associated fibroblasts (CAFs) not only represent one of the most important members but also are the largest proportion of stroma cells by secreting extracellular matrix components [12]. CAFs originate from bone marrow mesenchymal stem cells, resident fibroblasts, cancer cells, or endothelial cells, which is still under investigation. Besides, CAFs can differentiate when stimulated by ROS and TGF-*β*1-dependent and TGF-*β*1-independent mechanisms [13]. It was reported that CAFs influenced the tumor growth and progression, especially invasion and metastasis, via the secretion of many kinds of cytokines such as vascular endothelial growth factor A (VEGFA), CXCL12, Interleukin 6 (IL-6), and the physical remodeling of the ECM [14]. Compared with the normal fibroblasts, CAFs are highly heterogeneous and overexpress markers associated with malignant features, such as the platelet-derived growth factor receptors (PDGFRs) and the membrane-bound gelatinase fibroblast activation protein [15, 16]. The hyperactivated fibroblasts have been shown to enhance cellular migration [13] and elevate proangiogenic cytokine signaling [17, 18] and also can regulate the plasticity of cancer stem cells [19], facilitate inflammation [20], and adjust metabolism of epithelial tumor cells [21] (Table 1).

CAFs show both tumor-suppressive and tumor-promoting activities, due to their high heterogeneity and plasticity [22]. A set of biomarkers, such as fibroblast activation protein *α* (FAP-*α*), alpha-smooth muscle actin (*α*-SMA), PDGFR*α*/*β*, and vimentin, are highly expressed in CAFs and have been widely used to identify and isolate CAF populations.

### 2.2. Immune Cells

The tumor milieu creates a prospective shell where tumor cells rapidly accumulate gene mutations and immune escape. Especially in the early stage of cancer, the immune response produced by immune cells in the TME has antitumoral characteristics [9]. NK cells, CD8^+^ cytotoxic T cells, M1 macrophages, T helper-1 cells, and antigen-presenting cells (APCs) act as tumor opponents and suppress tumor growth. Accumulated evidence indicates that TME consists of a myriad of protumoral immune cells, such as neutrophils, tumor-associated macrophages (TAMs), CD4^+^ T helper-2 cells, and regulatory T cells (Tregs), which are the essential parts shaping the immune suppression environment, enabling tumor cell survival and metastasis, furthermore promoting the evasion of the immune destruction [3] (Table 1).

CD8^+^ cytotoxic T cells induce apoptosis, necrosis, and growth arrest by releasing INF-*γ*; then, APCs phagocytosed the residual proteins of apoptotic cells, which were exposed to maturing lymphocytes in lymphoid organs [23]. In contrast, Tregs attenuate the proliferation of CD8^+^ cells, inhibit APCs and macrophages, and reduce the lytic activity of NK cells [24]. Recently, daclizumab, the anti-CD25 monoclonal antibody, has been considered to suppress Tregs and enhance antitumor response [25].

During the cancer development, myeloid-derived suppressor cells (MDSCs), including macrophages, dendritic cells (DCs), and myeloid precursors, play a role in promoting tumor progression and angiogenesis, via suppressing T cells and NK cells by producing cytokines such as IL-6, IL-10, and TGF-*β* and also differentiating into TAMs under hypoxic conditions [9]. Macrophages are classified into M1 (proinflammatory) and M2 (anti-inflammatory) subtypes. M1 macrophages characteristically secrete proinflammatory cytokines, including IL-1 and TNF-*α*; thus, they promote antitumor response. In contrast, M2 macrophages, called tumor-associated macrophages (TAMs), release immunosuppressive cytokines, such as IL-10, to facilitate tumorigenesis [26]. Besides, TAMs play a role in regulating the invasiveness of the tumor through exporting oncogenic miR-233 in extracellular vesicles (EVs) [27]. Recently, it was revealed that TAMs regulated aerobic glycolysis and apoptotic resistance of the malignant tumor via the EV transmission of HIF-1-*α*-stabilizing lncRNA (HISLA) [28].

### 2.3. Endothelial Cells

Endothelial cells in TME have also been considered to interact with cancer cells [11]. Recently, the concept of “angiocrine factors” has emerged, which are released from tumor endothelial cells, such as adhesion molecules and chemokines, and vital for tumor progression and metastasis [29]. EVs secreted from endothelial cells can uptake angiogenic cargoes, including CD106 and CD49a, therefore elevating angiogenesis ability [30] (Table 1). Intriguingly, tumor cells could stimulate endothelial cells to promote tube formation and vascular growth by secreting multiple factors such as basic fibroblast growth factors (bFGF) and vascular endothelial growth factor (VEGF), mostly via activation of Akt and NF-*κ*B pathways [31]. In glioma carcinoma, EVs secreting from cancer cells promote angiogenesis and metastasis through directly transferring RNA and proteins, such as EGFRvIII and TF/VIIa, into endothelial cells [32]. Studies investigate that the anticancer therapy targeting cytokine secretion of endothelial cells may be a new breakthrough for chemotherapeutic agents [11].

### 2.4. Mesenchymal Stem Cells (MSCs)

MSCs are mainly composed of stromal cells that reside in mesenchymal tissues such as the bone marrow, cartilage, and fat tissues [33]. MSCs can differentiate into multiple cell types, including osteocytes, chondrocytes, and adipocytes [34]. Moreover, MSCs form the premetastatic niche for tumor cells which can promote cancer cell quiescence and drug resistance [33]. More recently, MSCs have been shown to migrate towards inflammatory sites and incorporate into the tumor. It was shown that crosstalk between MSCs and cancer cells at multiple stages of cancer progression was crucial for tumor metastasis and promoting epithelial-to-mesenchymal transition [33]. It was reported that exosomes derived from cancer cells trigger tumor growth through induction of MSC differentiation into myofibroblasts by activating the SMAD signaling pathway [35] (Table 1).

### 2.5. Other Cell Types

Other cell types, such as adipocytes and NE cells, have gradually been regarded as important regulators of cancer development and a possible source of prognostic indicators for cancer patients.

Since the foundation of leptin in 1994, adipose tissue is considered as a functional and secreted endocrine organ [36]. Adipose tissue participates in cancer growth and progression via secreting more than 50 various cytokines, hormone-like factors, and chemokines and reprogramming proinflammatory microenvironment [3]. Recent evidence highlights adipocytes as a key component of breast cancer progression [37]. In addition, it was reported that when cancer cells were cocultured with adipocytes, the breast cells exhibited an aggressive phenotype via cancer-secreted exosomal miR-15, which also acts as an oncogenic signal to reprogram cell metabolism [38] (Table 1).

NE cells are spread throughout the normal organism and exist in tissues including the hypothalamus, anterior pituitary gland, thymus, thyroid gland (calcitonin-secreting cells), breast, and pancreatic islets [3]. NE cells from almost all malignant tumors exert proproliferation function by generating and secreting multiple neurotransmitters, such as chromogranin A (CgA), chromophilic polypeptide, and vasoactive polypeptide, eventually influencing tumor progression [39]. Extensive evidence has proven that NE cells regulate the function of the immune system, such as influencing NK cell viability and prometastasis ability through neurotransmitters, therefore adjusting the cancer development [40] (Table 1).

### 2.6. Vascular and Lymphatic Networks

In 1971, Judah Folkman proposed a theory that all malignant tumors were angiogenesis-dependent [41]. Angiogenesis is a biological process in which new capillary blood vessels grow from the preexisting vasculature environment, in response to the interaction between tumor cells and endothelial cells, as well as ECM components and other growth factors [42]. Tumor blood vessels in TME provide fresh oxygen and nutrition support for tumor tissues and help cancer cells move into the blood stream and spread to distant sites (Table 1). Certain proangiogenic molecules such as VEGF, TGF-*α* and TGF-*β*, epidermal growth factor, and antiangiogenic regulators including angiostatin, endostatin, IL-12, thrombospondin-1 (TSP-1), tissue inhibitors of metalloproteinases (TIMPs), and interferon-*α*, interferon-*β*, and interferon-*γ* are all well-studied [43].

Hypoxia is the primary factor that drives tumor angiogenesis and causes the upregulation of VEGF [43]. Moreover, the lymphatic network impacts heavily on cancer progression and prognosis, which may represent a possible route for systemic dissemination of cancer cells [2]. In particular, lymphatic vessels around the tumor tissue provide a traffic link between the lymph nodes and the primary tumor. Thus, collateral lymphatic vessels can also provide the diminution in lymphatic capacity when lymphatic vessels are obstructed [3] (Table 1). The vascular and lymphatic networks help cancer cells escape immune surveillance from two categories: the lymphatic microenvironment directly weakens the normal function of immune cells and the remolding of vascular endothelial cells indirectly affects the access of immune cells into lymph nodes [3]. For example, MDSCs and immature DCs can inhibit the normal function of T cells in the sentinel lymph nodes to eliminate the immune response. In addition, when cancer cells transfer to an abnormal environment, CD4^+^ T and CD8^+^ T cells may help tumor cells escape immune surveillance [3].

Currently, targeting angiogenesis has become a hot topic in the research of cancer therapeutics and has achieved a good clinical efficacy [3]. Nonetheless, the early antiangiogenesis therapy failed with huge disappointment of the scientific community. Tumor vessels possessed abnormal structures with a chaotic blood flow and vessel leakiness, as a result of endothelial cell disorganization, fewer pericyte covering, and irregular basement membrane. The vascular distribution in tumor tissues is heterogeneous, causing the impaired tumor perfusion and a hypoxic microenvironment, which reduced the diffusion of chemotherapeutic drugs. Moreover, induced by this therapy, different cancers developed multiple signaling pathways, which lead to worse outcomes in accordance with drug resistance and tumor metastasis [44]. Accumulating evidence now suggests that the normalization of the tumor vasculature can limit tumor cell invasiveness and enhance the effectiveness of anticancer therapy, by restoring proper tumor perfusion and improving oxygenation [45]. For example, targeting VEGF and VEGFR signaling has successfully induced vascular normalization in tumors by pruning unnecessary immature vessels, improving vessel perfusion. Other targeted factors, such as angiopoietin families, regulator of G-protein signaling 5, and platelet-derived growth factors, may be blocked and contribute to the vessel normalization [44]. What is more, the potential functional importance of vascular mimicry (VM) has recently been highlighted in differentiated malignancies by several studies, an alternative route exploited by tumor cells to sustain tumor perfusion and thus growth, even when angiogenesis is lacking or inhibited. Maniotis reported that VM was an endothelial-independent vascular channel that contained red blood cells, formed with highly aggressive and metastatic cancer cells in 1999. The structure of VM was a lack of endothelial cells in the internal lining, with CD34 immunohistochemical staining negative/Periodic acid-Schiff (PAS) histochemical staining positive [46]. Furthermore, CSCs possess the highest plasticity and may transdifferentiate to ECs by EMT induction. Several studies also demonstrated that VM capacity correlated with CD133 CSC marker expression in many tumors. VE-cadherin, EphA2, FAK, PI3K/Akt, MMPs, VEGF and VEGFR1/2, HIF-1, and other relevant molecules are involved in VM formation [47]. Except western medicines, such as thalidomide, zoledronic acid (ZA), and celecoxib, traditional Chinese medicine curcumin was observed to inhibit tumor growth and VM formation through downregulating the EphA2/PI3K/MMP pathway [48]. Accumulated studies revealed that targeting VM-related molecules with novel antiangiogenic therapies to inhibit VM formation was a promising therapeutic target.

### 2.7. Extracellular Vesicles (EVs)

EVs, which are membrane-wrapped vesicles, including exosomes, microvesicles, and apoptotic bodies, are ubiquitous in human tissues and the circulation system [49]. EVs have emerged as critical mediators of the distant communication between the tumor and the TME cells by carrying multiple biologically active molecules, which can promote cancer initiation and progression [4, 8].

The specific functions of EVs among multiple cancers are vastly different, depending on their biogenesis and cargoes (proteins, lipids, messenger RNAs, micro-RNAs, long noncoding RNAs, mitochondrial DNAs, and other nucleic acids) [49]. Transfer of these components from cancer cells to TME helps to establish a niche for cancer survival and mobility. Tumor cell-derived EVs have been shown to regulate key signaling pathways in tumor and TME, which can also reprogram stromal cells to generate a cancer cell niche [8]. Meanwhile, stromal cell-derived EVs are capable of affecting the proliferation, drug resistance, and stem cell properties of cancer cells [8]. EVs also mediated the crosstalk between cancer cells and diverse TME cells such as adipocytes, fibroblasts, bone marrow cells, and immune cells [11]. Emerging evidence suggests that tumor cells secrete more EVs than normal cells. More importantly, the content of EVs derived from different cell types showed distinct content profiles, which make it an emerging category of disease markers [50]. Since EVs could be easily obtained from blood, urine, and saliva, they could serve as promising biomarkers for early cancer diagnosis. Recently, large oncosomes, the newly identified EVs, have been found correlating with tumor progression in human and mouse models [51]. In particular, the newest finding shows that circulating small extracellular vesicle- (sEV-) derived miRNAs have a greater perspective effort for early diagnosis of colon cancer, compared with plasma total miRNAs [52]. As the sensitivity of EV isolation techniques improves, the specific cargo inside EVs allows them to serve as cell-free biomarkers in cancer diagnosis and targets to cancer therapy resistance [50] (Table 1).

### 2.8. Extracellular Matrix (ECM)

The ECM is a noncellular three-dimensional network, classically composed of collagen, elastin, fibronectin, proteoglycans, laminins, and other glycoproteins [3]. Each matrix component binds each other with cell adhesion receptors, forming the complex macromolecular network. Cell surface receptors transduce signaling pathways into cells from ECM, contributing to varieties of tumor biological behaviors, such as survival, migration, differentiation, and metabolism [53]. Most ECM proteins experience a complex posttranslational modification, such as glycosylation, sheering, and covalent crosslinking. Besides, lysyl oxidase (LOX) and matrix metalloproteinases (MMPs) are major modulations for ECM [54]. Recent studies suggest that ECM proteins, such as Asporin, may not only have extracellular functions but also have essential intracellular functions to promote tumor proliferation [55, 56]. Emerging evidence indicates that the heterogeneity of ECM plays a crucial role in tumor proliferation by providing cells with sustaining growth signals, evading growth suppressors, and resisting cell death, also in tumor angiogenesis, invasion, and metastasis [57] (Table 1).

The MMP family is a class of proteolytic enzymes that degrade components of the ECM. High levels of MMP expression are correlated with poor prognosis in multiple malignancies, including MMP-1, MMP-7, MMP-9, MMP-11, and MMP-13 [58]. The current study also identifies that the high expression of MMP-19 and MMP-20 is associated with the poor prognosis of ovarian cancer [59]. Indeed, due to their abilities of cleaving, degrading, and rearranging ECM molecules, MMPs play a critical role in proteolysis and detachment of tumor cells from the ECM, what tumor cells need to breach vascular barriers and move into the blood stream and spread to distant organs, also resulting in cancer stem cell formation and metastasis [60]. Recent study demonstrates that MMP-10 is required for maintenance of the lung cancer stem cell with a loss of stem cell surface marker expression and stimulates tumor initiation and metastatic ability [61]. Membrane type 1 MMP is a cell surface proteinase, which not only is involved in cancer survival and invasion but also helps exhibit cancer stem cell-like characteristics, including self-renewal ability, low proliferation, resistance to chemo- and radiotherapy, and resistance to apoptosis [62]. Currently, clinical trials targeting MMPs were not successful for the difference of tumor growth environment between the human and the murine; therefore, next more research needs to perform trials in early cancers, identifying effective biomarkers of enzymatic inhibition for clinical success [58].

## 3. Physical and Chemical Characteristics of TME

The tumor microenvironment shows profound differences from human normal tissues in terms of physiological characteristics at the cellular and tissue levels. These functional parameters include extracellular pH, hypoxia, elevated interstitial fluid pressure, and cancer-associated fibrosis between TME and normal intracellular environment. These factors are closely linked and related to every step in the progression, metastasis, and metabolism of tumors. Changes in the complex environment are always in a dynamic process and provide amplified growth surroundings and material conditions for tumor progression, immunosuppression, and treatment resistance.

### 3.1. Extracellular pH

Acidification of the TME plays an established role in tumor progression and provides a hostile milieu which advantages tumor survival and growth compared to nontumoral cancers. Even when oxygen supply is sufficient, tumor cells can create a low pH environment through increased glycolytic activity (known as the Warburg effect) and the production of monocarboxylated transporter- (MCT-) 4 and sodium-proton transporters that normalize intracellular pH [6, 63–65]. During this process, tumor cells accumulate high levels of metabolism productions and low glucose concentrations [66]. Simultaneously, many tumors show pronounced extracellular acidity with pH values even lower than 6.5 [67]. It is generally believed that the formation of acidification of TME involves two parts: lactic acid produced by glycolytic metabolism and CO_2_ by respiration. Besides, both the poor blood perfusion and the lack of functional lymphatic vessels limit the acid metabolism substances from TME [68]. Additionally, tumor cells possess all enzyme systems to adjust to the acid environment that plays a crucial role in cancer progression [6, 64].

It is reported that acidic regions are not only restricted to hypoxic areas but overlapped at the tumor-stroma interface which plays a crucial role in tumor proliferation and invasion [69]. Some people explained that neighbor normal stromal cells can absorb the large amount of lactic acid released from tumor cells to regenerate pyruvate and restrict extracellular overacidification [70]. Moreover, the association between tumor microenvironment acidosis and tumor invasion is well understood. The lactic acid produced by glycolysis promotes the synthesis of hypoxanthine and the expression of its transmembrane receptor CD44. The binding of hypoxanthine and CD44 can reduce the adhesion between tumor cells [71]. Also, acidosis-driven adaptation promotes immune escape and may offer a broad panel of therapeutic targets [72]. From another aspect, although tumor cells mainly acquire fast energy through aerobic glycolysis, recent study suggested that cancer cells under lactic acidosis switch from Warburg effect back to oxidative phosphorylation (OXPHOS) phenotype, through inhibiting the expression of HIF-1*α* and thus leading to aggressive phenotype. The ability that tumor cells are plastic and can shift metabolic phenotypes to adjust the changeable microenvironment gives a selective advantage to cancer cells upon lactic acidosis [73].

Some evidences indicate that extracellular acidosis confers a useful and adequate niche to dormant tumor cells for supporting disseminated tumor cell survival and metastasis formation and therefore sustaining a resistant chemo- and radiotherapy phenotype [74]. On the other hand, the recent finding implies that the acidic microenvironment promotes anoikis resistance, through mTOR/NF-*κ*B signaling and adds new possible mechanisms to metastatic spread of solid tumors [75]. In conclusion, the distinct and changeable energy metabolic phenotype in cancer cells provides multiple potential opportunity for treatment.

### 3.2. Hypoxia

It is well known that tumor hypoxia is an important microenvironment factor that causes cancer development and resistance to cancer treatment. Approximately 60% of human tumors show distinct levels of hypoxia and even anoxia in tumor tissues. It is reported that adaption to the hypoxia environment is the foundation for cancer tissues' survival and growth. Indeed, abnormal and dysfunctional tumor blood vessels are incapable of restoring oxygenation because of the loss in the transportation of oxygen, therefore perpetuating hypoxia, which in turn will promote cancer progression, metastasis, and resistance to antitumor therapies [5].

Accurate regulation of oxygen homeostasis is essential for cell death and survival. Hypoxia-inducible factors (HIFs) are considered to be the executors of the response to hypoxia [76]. There is ample evidence of the positive correlation between HIFs and tumor progression, metastasis, and poor prognosis [77]. Interestingly, HIFs do not directly sense variations to oxygen tension (pO2) but are regulated by prolyl-4-hydroxylase 2 (PHD2) in response to oxygen availability [78]. In normoxic conditions, HIF-1*α* is negatively regulated by activated PHD2 in the presence of O_2_, Fe^2+^, and 2-OG at the Pro402 and Pro564 residues of the C terminus [76, 79]. Besides, once HIF-1*α* is hydroxylated by PHD2 at the proline residues, it is further captured by pVHL and ultimately targets its proteosome polyubiquitination [80]. By contrast, hypoxia results in the inhibition of PHD2 activation, causing accumulation of HIF-1*α* and then dimerization with the HIF-1*β* subunit. Consequently, many HIF-mediated proangiogenic genes including the vascular endothelial growth factor (VEGF) and fibroblast growth factor-2 (FGF2) are activated, which enhance the metabolism of glucose and fatty acids, metastasis, invasiveness, and angiogenesis [81]. There is more evidence shown that PHD2 silencing in cancer cells can exert both pro- and antitumoral effects, depending on the cellular context. On the one hand, PHD2 promotes metastasis through activation of CAFs and inactive PHD2 inhibits proliferation and growth in breast cancer [82], stroma and bone marrow-derived cells [83], lung carcinoma [84], B-cell lymphomas [85], hepatocellular carcinoma [86], and head and neck squamous cell carcinoma [87]. On the other hand, there is some evidence for the antitumoral effect of PHD2 in gastric adenocarcinoma [87], non-small-cell lung cancer [87], and prostate cancer [88]. Taken together, PHD2 may have an important role in regulating HIF and cancer progression and have been considered as a potential therapeutic target in treating cancers.

### 3.3. Interstitial Fluid Pressure (IFP)

Abnormal blood and lymphatic vessels create a hostile TME with hypoxia, low pH, and elevated interstitial fluid pressure (IFP). The high IFP in the TME is considered as the key barrier commonly seen in solid tumors that can impede drug delivery to tumors. It is believed that elevated tumor IFP is from high cell density, increased vascular permeability, impaired venous or lymphatic drainage, and abnormal ECM [7]. In the limited space of TME, abnormally increased cancer cells make mechanical compression of lymphatic vessels and blood vessels, resulting in poor lymphatic drainage and blood flow, further causing the number of functional lymphatic vessel decreases and abnormal vascular structures [89].

Excess fluid leaks from the vasculature into the interstitium, where it accumulates and distends the elastic ECM, elevate IFP compared to normal tissues. The IFP values of 5-40 mmHg in solid malignant tumors are reported, whereas in most normal tissues, it is ranging from -3 to +3 mmHg. The increased IFP causes a positive pressure gradient, which is a driving force for a connective transport back into the capillaries or to adjacent regions with low IFP [89]. Therefore, the high IFP profoundly reduces drug delivery efficacy due to a drop of convection between the intravascular and extravascular spaces and thus limiting drug distribution into the TME. It is reported that the increased IFP is associated with a poor prognosis in many solid tumors, such as melanoma and cervical cancer [90]. Also, it is demonstrated that reducing the IFP in tumors via treating tumor-burdened mice with a vascular disrupting agent correlates well with tumor size reduction [91]. Thus, by targeting components that create high IFP in the TME, drug delivery to tumors can be improved.

### 3.4. Tumor Fibrosis

Tumor fibrosis derived from the excess deposition of the crosslinked collagen matrix by CAFs, MSCs, stellate cells, and fibrocytes [92]. Briefly, chronic inflammation results in cancer fibrosis. Once tissue injures, this “nonhealing wound” is created. Normal tissue fibrosis restraints cancer initiation and invasion. However, cancer-associated fibrosis promotes cancer cell crosstalk and progression and is differently regulated in terms of four reasons: stromal source, stromal reprogramming under cancer mediation, fibrosis subtype, and the impact of other TME components [93]. In in vivo and vitro studies, chemotherapy and radiation therapy are also drivers of fibrosis via generating the hypoxia microenvironment and activating the immune system [93]. The impact of cancer fibrosis on cancer behavior is controversial. For example, undergoing cancer education, normal tissue MSCs are inverted into cancer-associated MSCs and communicate with cancer cells via forming a positive feedback loop, BMP4:HH, to promote cancer growth and drug resistance and enrich them stem cell-like pool [94]. Besides, MSC residents in tumors are considered favoring immune evasion. It was evidenced that MSCs secreted immunosuppressive factors including nitric oxide, IL-4, TGF-*β*, and several soluble program death ligands 1 and 2 to suppress CD4^+^ T cells and promote Treg formation [95]. Hedgehog is a critical fibrosis signaling pathway [93]. As we depicted before, tumor fibrosis is a positive factor for cancer progression. A recent study suppressed fibrosis by knocking down the Hedgehog signaling pathway, leading to more aggressive and poorly differentiated tumors [96]. Currently, the antifibrosis drugs, pirfenidone and nintedanib, via clinically combining with chemotherapy treatment, have demonstrated a survival benefit [92]. It is important to realize the heterogeneity of TME, and cancer-associated fibrosis evolves a dual function during cancer progression. We believe that tumor fibrosis has the potential to be a future therapeutic target for cancer.

## 4. Contributions to the Early Diagnosis of Cancers

Noninvasive molecular imaging is essential for exhibiting visualization of molecular and cellular components and provides a further understanding of cancer pathogenesis and cell-to-cell interaction. Researchers have been dedicated to finding new biomarkers and diagnostic methods for the estimation and continuous measurement of cancer treatment responses in the TME.

Recently, novel specific molecular probes detecting components of TME have been investigated in vitro and in vivo. Moreover, along with the development of molecular therapy and next-generation sequencing, the studies on CTC (circulating tumor cell) and cfDNA (circulating free DNA) have been the hit of oncology. These approaches were expected to facilitate the implementation of individualized and precise treatment of cancer patients.

### 4.1. Molecular Imaging of TME

Conventional imaging technologies include three forms: radionuclide-driven approaches including positron emission tomography (PET) and single-photon emission computed tomography (SPECT), magnetic resonance imaging (MRI), and optical imaging. Labeling strategies for cell tracking or targeting of effector molecules in the TME enable visualization of tumor-associated inflammation, hypoxia, and pH alteration, as well as integrins and enzymes [9]. For example, to visualize phagocytosis of TAMs, researchers invented mannosylated liposomes loaded with ^64^Cu which used PET imaging for observation after being taken by TAMs in a mouse model of pulmonary tumor [97]. Besides, ^89^Zr-modified reconstituted high-density lipoprotein (HDL) is designed as a label for PET imaging of TAMs for higher specificity [98]. For MRI cell tracking technologies, by injecting superparamagnetic iron oxide nanoparticles (SPIOs), TAMs are systemically assessed for local accumulation during tumor development. As introduced in a preclinical study, ^99m^Tc-labeled single-domain antimacrophage mannose receptor helps TAMs detected by SPECT in breast and lung tumors [99]. Moreover, injection of luciferase-expressing murine macrophages helps in vivo cell tracking in a colon cancer murine model, though injected cells influence mouse tumor growth response to dexamethasone [100].

Besides tracking and monitoring tumor-associated inflammation, there are different modes of imaging probes for targeting hypoxia and pH changes in the TME. PET/SPECT tracers for imaging hypoxia are made successful in the clinic. The most widely utilized hypoxia imaging PET/SPECT tracer is 1-(2-nitroimidazolyl)-3-[^18^F]fluoro-2-propanol (FMISO) [101], which was found to provide better quality images of the hypoxia tumor area in humans at 4 hours, with an accurate reflection of HIF-a and VEGF [102]. ^18^F-labeled PET hypoxia imaging is also examined for detecting changes before and during treatment and has a promising prognostic value for evaluating TME changes after cancer therapy [103–105]. Besides, optical imaging of hypoxia in the TME has been investigated with multiple probes, including fluorescent, phosphorescent, and Förster energy transfer (FRET) off-on probes [106, 107].

In the early 1980s, tumor pH measurements were detected by pH electrodes with low sensitivity. Currently, various pH probes for MRS and MRI imaging use the physical properties of acidic protons, and the mainly known technique to measure the tumor region is acidoCEST (acid chemical exchange saturation transfer) with iopromide. Recently, it was reported that there are two novel approaches to imaging the tumor pH region. PET imaging of FDG-glycosylamine (FDG-amine 4) can only detect the tumor having an acidic microenvironment [108]. pH (low) insertion peptides (pHLIPs) have gained increased application in imaging the TME for them localizing and detecting tumor tissues compared to normal tissues [109, 110].

Nowadays, molecular imaging has been further investigated for possible clinical applications, especially assistance in surgery. Multiphoton imaging for collagen imaging in early gastric cancer revealed the role of collagen in TME and helps develop a prediction model for lymph node metastasis based on collagen signature [111]. Nevertheless, imaging in vivo contributes to representing a real-time visualization of tumor biology and helps better monitoring of therapeutic effects.

### 4.2. High-Throughput Multiplex Immunohistochemical Imaging (mIHC) of the TME

Conventional tissue imaging with HE staining and immunohistochemistry is considered as a key for the diagnosis of the cancer subtype and malignant degree. Recently, a high-throughput mIHC technology based on brightfield IHC was developed for better visualization of TME with imaging various immune harboring complex immunophenotypes and further for the subcellular localization of target molecules [112].

### 4.3. Nanostructured Probes

Comparing with massively established parallel DNA sequencing, high-throughput protein profiling remains challenging. A recent study invented a nanostructured barcode for accurately classifying the subtypes of breast cancer and identifying subcellular spatial markers of tumor aggressiveness [113]. Molecular imaging probes for tumor diagnosis based on specific biomarkers usually have a limited sensitivity. Comparing with normal tissues, low pH and hypoxia can be well utilized to identify tumor tissues. A kind of near-infrared polyconjugated iridium complex was designed to differentiate tumor and normal tissues, via detecting the acidity and oxygen content in the solid tumor. These optical probes were activated only in the TME and utilized for detecting tumors minimum 1 mm in diameter, so they highly improved the sensitivity of cancer detection [114]. Another research invented an exogenously administered tumor-penetrating nanosensor, which sheds peptide fragments, detected in the urine, in response to a tumor-specific protease, MMP9. Although there is a difficulty that normal tissue expresses a little of MMP9, the mouse model results predicted that this probe can help identify human ovarian cancer up to five months earlier than current biomarker detections [115].

### 4.4. Liquid Biopsy

Accumulating evidence suggested that the potent clinical applications of circulating tumor cells (CTCs), cell-free DNA (cfDNA), circulating RNAs (miRNA, lncRNAs, and mRNAs), and exosomes have emerged as new biomarkers for noninvasive cancer diagnosis. Liquid biopsy from the peripheral blood sample of cancer patients is less invasive and inexpensive when compared with tissue biopsy. Sampling from patients can be easily acquired and repeated to monitor changes during cancer treatment.

CTCs are generally recognized shed into peripheral blood from cancer in situ and eventually establish multiple metastatic tumors in other organs. Despite their rarity, CellSearch™ is currently the only assay for the identification and characterization of CTCs in the clinical application [10]. Although CTCs in peripheral blood have been proved to be elevated in the bladder and rectal cancer patients with advanced stage and were associated with poor prognosis, they are not fully accepted for guiding treatment decisions. Recently, researchers are struggling with analyzing CTCs' content, such as microRNAs, for investigating new biomarkers [116].

Circulating cfDNA is a short fragment double-stranded DNA, originating mainly from apoptotic or necrotic cell death [117]. cfDNA released by tumors carries tumor-specific alterations such as copy number variation, point mutations, and DNA methylation. Currently, digital PCR has been a very sensitive tool for detecting point mutations and methylated genes in cfDNA. More recently, targeted and whole-genome sequencing technologies are increasingly applied for cfDNA analysis [118]. It was revealed that the level of cfDNA in the blood from cancer patients was observed frequently increased than normal patients [119]. Moreover, plasma cfDNA has been identified as an early prognostic and predictive biomarker for cancer patients, including melanoma [117], non-small-cell lung cancer [120], colorectal cancer [121], hepatocellular carcinoma [122], and prostate cancer [123]. A critical limitation of cfDNA testing is its short half-life so that quick sampling in a short time is of vital importance. Tumor-specific mutations are only detected in 0.01% of total cfDNA, which makes the detection of rare variants still challenging.

Circulating miRNAs have also been identified as potential cancer biomarkers. Many studies have reported circulating miR-210 as a diagnostic marker for rectal cancer, miR-126 for bladder cancer, and miR-21 for prostate cancer. Although circulating mRNAs were first discovered in the 1990s, their lack of stability and interindividual variability restrained the wide application. Since the protective role of EVs' contents such as long-chain RNAs gradually revealed, the application of long-chain RNAs as a novel biomarker has recently attracted much more attention than ever. Recently, several mRNAs packaged into circulating EVs, such as AR-V7 in prostate cancer and hTERT in bladder and prostate cancers, were considered to be promising biomarkers [10]. The most notable lncRNA is prostate cancer antigen 3 (PCN3), as a specific biomarker for prostate cancer. More recently, researchers are combining single circulating marker into one multimarker test to improve the accuracy of diagnosis.

It is worth mentioning that plasma EV detection has emerged as a novel approach in liquid biopsy. EVs, as we have mentioned before, play a critical role in intercellular communication by transferring biologically active molecules. Small EVs, most of which were considered to be exosomes, isolated from the plasma of cancer patients present a different content profile as compared to normal subjects [52]. Recent studies reported that exosomal miRNAs (such as miR-34a, miR-148a), lncRNAs (such as ARSR, HOTAIR, HOX-AS-2, ANRIL, and linc-RoR), and serum MDR-1, MDR-3, and PABP4 proteins have potential to serve as predictive biomarkers [124–126]. The most important limitation of the application of plasma exosomes as a biomarker is the lack of a robust isolation method with both high recovery and high specificity. However, with the progress of EV methodology and establishment of consensus on EV studies, we believed that soon, an exosomal biomarker would be one of the most promising new biomarker categories applicated in the clinical practice.

## 5. Conclusions

In summary, we introduced the complex network of TME, ranging from cellular components, such as fibroblasts, immune cells, endothelial cells, vascular network, and EVs to the metabolic environment including acidosis, hypoxia, interstitial fluid pressure, and tumor fibrosis. Although we included a large amount of information in our study, many crucial biochemical processes in TME, such as the educated regulation between normal cells and cancer cells, remain unknown.

Although there are various approaches for specific detection of TME components, such as molecular imaging, nanostructured probe, and liquid biopsy, most of them are still not ready for clinical use. Nonetheless, with the growing interest in basic and translational studies of TME, the more information we acquire, the closer we are to their clinical application.

## Figures and Tables

**Table 1:** Components, functions, and classifications of TME.

Component	Function	Classification
Cancer-associated fibroblasts (CAFs)	Sustaining proliferative signaling; activating angiogenesis and metastasis; tumor-promoting inflammation; evading immune destruction; reprograming cellular metabolism; promoting genome instability and mutation.	Tumor promoting; less known of tumor inhibiting; abundant in TME; commonly used markers including *α*-SMA, FAP-*α*, FSP-1/S100A4, and PDGFR*β*; the origin of CAFs is not clear, and CAFs can differentiate stimulation by ROS and TGF-*β*1-dependent and TGF-*β*1-independent mechanisms.
Immune cells		
Neutrophils	Enhancement of angiogenesis and metastasis; associated with poor prognosis.	Tumor promoting (N2); tumor inhibiting (N1); increased levels in the colon, stomach, and lung cancer patients.
Tumor-associated macrophages (TAMs)	Promoting degradation of the extracellular matrix; aiding the expansion of inflammatory cytokines, such as TNF-*β*; enhancement of angiogenesis and remodeling.	Tumor promoting (M2); tumor inhibiting (M1); the major protumoral component in TME; the first nonneoplastic cells infiltrating the tumor; attracted by chemokines secreted by both malignant and stromal cells.
CD8^+^ cytotoxic T cells (CTL)	Induce apoptosis, necrosis, and growth arrest by releasing INF-*γ* and other cytotoxic cytokines; establishing an antitumor environment.	Tumor inhibiting; the major antitumoral component in TME.
Regulatory T cells (Tregs)	Secreting cytokines such as IL-10, TGF-*β*; establishing an immunosuppressive environment; associated with poor prognosis.	Tumor promoting; promoting tumor maintenance.
Myeloid-derived suppressor cells (MDSCs)	Associated with tumor progression and neoangiogenesis; suppressing T cells and NK cells; differentiating into TAMs under hypoxic conditions.	Tumor promoting; increased in almost all patients/animals with cancer; including premature granulocytes, macrophages, dendritic cells, and myeloid precursors.
Mesenchymal stem cells (MSCs)	Differentiating into mesenchymal tissues such as bone, cartilage, and fat tissues, vasculogenic mimicry; forming the premetastatic niche; promoting cancer initiation and malignancy.	Tumor promoting; the major component of stromal cells in TME.
Endothelial cells	Consisting of tumor blood vessels; secreting angiocrine factors such as adhesion molecules; intercommunicating with tumor cells via secreting EVs including CD106, CD49a.	Tumor promoting.
Adipocytes	Regulating the balance of systematic energy and metabolism; secreting exosomes, cytokines, chemokines, and hormones; promoting cancer progression.	Tumor promoting.
Neuroendocrine cells (NE cells)	Promoting proliferative signaling; secreting neurotransmitters, including CgA, chromophilic and vasoactive polypeptide; regulating NK cell migration and toxicity ability.	Tumor promoting.
Vascular network	Providing oxygen, clearing carbon dioxide, and metabolizing wastes; providing nutrition support for cancer cells; promoting angiogenesis and metastasis.	Tumor promoting; all malignant tumors are angiogenesis-dependent.
Lymph vessels	Helping immune cell avoid immunity and dissemination; providing a physical link between lymph nodes and tumor.	Tumor promoting.
Extracellular vesicles (EVs)	Carrying biologically active molecules such as proteins, miRNAs, and lncRNAs from donor cell to recipient cell; regulating key signaling pathways, proliferation, drug resistance, and stemness; reprogramming stromal cells to create a niche for survival.	Tumor promoting; tumor inhibiting; membrane-wrapped vesicles including exosomes, microvesicles, and apoptotic bodies; as a critical mediator between tumor and the TME.
Extracellular matrix (ECM)	Forming the complex macromolecular network; controlling cancer invasion and metastasis, angiogenesis; contribution to growth and proliferation signaling, inhibiting cancer apoptosis.	Tumor promoting; a noncellular three-dimensional network including collagen, elastin, fibronectin, proteoglycans, laminins, and glycoproteins.
